# Multiparametric Circulating Tumor Cell Analysis to Select Targeted Therapies for Breast Cancer Patients

**DOI:** 10.3390/cancers13236004

**Published:** 2021-11-29

**Authors:** André Franken, Bianca Behrens, Florian Reinhardt, Liwen Yang, Mahdi Rivandi, Francesco Marass, Bernadette Jaeger, Natalia Krawczyk, Jan-Philipp Cieslik, Ellen Honisch, Hannah Asperger, Emmanuelle Jeannot, Charlotte Proudhon, Niko Beerenwinkel, Natali Schölermann, Irene Esposito, Frederic Dietzel, Nikolas H. Stoecklein, Dieter Niederacher, Tanja Fehm, Hans Neubauer

**Affiliations:** 1 Department of Obstetrics and Gynecology, University Hospital and Medical Faculty of the Heinrich-Heine University Duesseldorf, 40225 Duesseldorf, Germany; Andre.franken@med.uni-duesseldorf.de (A.F.); Florian.Reinhardt@med.uni-duesseldorf.de (F.R.); Liwen.Yang@med.uni-duesseldorf.de (L.Y.); Mahdi.Rivandi@med.uni-duesseldorf.de (M.R.); bernadette.jaeger@med.uni-duesseldorf.de (B.J.); natalia.krawczyk@med.uni-duesseldorf.de (N.K.); Jan-Philipp.Cieslik@uni-duesseldorf.de (J.-P.C.); Ellen.Honisch@med.uni-duesseldorf.de (E.H.); Hannah.Asperger@uni-duesseldorf.de (H.A.); Niederac@med.uni-duesseldorf.de (D.N.); Tanja.Fehm@med.uni-duesseldorf.de (T.F.); 2 General, Visceral and Pediatric Surgery, University Hospital and Medical Faculty of the Heinrich Heine University Duesseldorf, 40225 Duesseldorf, Germany; Bianca.Behrens@med.uni-duesseldorf.de (B.B.); nikolas.stoecklein@hhu.de (N.H.S.); 3 Department of Biosystems Science and Engineering, ETH Zurich, 4058 Basel, Switzerland; francesco.marass@bsse.ethz.ch (F.M.); niko.beerenwinkel@bsse.ethz.ch (N.B.); 4 Swiss Institute of Bioinformatics, 4058 Basel, Switzerland; 5 Circulating Tumor Biomarkers Laboratory, SiRIC, Institut Curie, PSL Research University, 75005 Paris, France; emmanuelle.jeannot@curie.fr; 6 Department of Pathology, Institut Curie, 75005 Paris, France; 7 Institut Curie, INSERM U934/CNRS UMR3215, PSL Research University, 75248 Paris, France; Charlotte.Proudhon@curie.fr; 8 Frauenarztpraxis Dr. med. Natali Schölermann, 42781 Haan, Germany; natali.schoelermann@gmx.de; 9 Institute of Pathology, University Hospital and Medical Faculty of the Heinrich-Heine University Duesseldorf, 40225 Duesseldorf, Germany; Irene.Esposito@med.uni-duesseldorf.de; 10 Department of Diagnostic and Interventional Radiology, University Hospital and Medical Faculty of the Heinrich-Heine University Duesseldorf, 40225 Duesseldorf, Germany; Frederic.Dietzel@med.uni-duesseldorf.de

**Keywords:** AKT1, breast cancer, circulating tumor cells, targeted therapy, whole exome sequencing

## Abstract

**Simple Summary:**

Liquid biopsies may act as a dynamic tool for identification of targets for precision therapy while circumventing limitations of tissue biopsies. In opposite to most liquid biopsy-related studies that analyze limited patient material for only one parameter, this study is based on a longitudinal and multiparametric analysis of circulating tumor cells (CTCs). A metastatic breast cancer patient was followed over a period of three years and analyses of the genome, RNA profiling, and in vitro drug testing on cultured CTCs were performed in a unique manner. We show that combining the strengths of multiple technologies for analysis yielded maximum information on the ongoing disease and, eventually, allowed choosing an effective therapy, which led to a massive reduction in CTC numbers. This approach provides a concept for future detailed longitudinal and multiparametric CTC analyses.

**Abstract:**

Background: The analysis of liquid biopsies, e.g., circulating tumor cells (CTCs) is an appealing diagnostic concept for targeted therapy selection. In this proof-of-concept study, we aimed to perform multiparametric analyses of CTCs to select targeted therapies for metastatic breast cancer patients. Methods: First, CTCs of five metastatic breast cancer patients were analyzed by whole exome sequencing (WES). Based on the results, one patient was selected and monitored by longitudinal and multiparametric liquid biopsy analyses over more than three years, including WES, RNA profiling, and in vitro drug testing of CTCs. Results: Mutations addressable by targeted therapies were detected in all patients, including mutations that were not detected in biopsies of the primary tumor. For the index patient, the clonal evolution of the tumor cells was retraced and resistance mechanisms were identified. The AKT1 E17K mutation was uncovered as the driver of the metastatic process. Drug testing on the patient’s CTCs confirmed the efficacy of drugs targeting the AKT1 pathway. During a targeted therapy chosen based on the CTC characterization and including the mTOR inhibitor everolimus, CTC numbers dropped by 97.3% and the disease remained stable as determined by computer tomography/magnetic resonance imaging. Conclusion: These results illustrate the strength of a multiparametric CTC analysis to choose and validate targeted therapies to optimize cancer treatment in the future. Furthermore, from a scientific point of view, such studies promote the understanding of the biology of CTCs during different treatment regimens.

## 1. Introduction

In the last decades, targeted therapy has become the preferred treatment approach in many cancers. However, obtaining information to choose targeted therapies is challenging: biopsies of recurrent or metastatic lesions are invasive and can often not be performed when clinical conditions have worsened or when a tumor is inaccessible [1]. Furthermore, the genomic profile of biopsy tissues provides a picture limited to a single point in space and time, and may thus under-represent intratumoral heterogeneity [2]. Such factors limit the predictive utility of tissue biopsies, worsened still by the continuous evolution of the tumor cells in response to endogenous and exogenous selective pressures [3]. To overcome this challenge, the idea of deriving information about the primary tumor (PT) or metastatic lesions from liquid biopsies, which may act as a dynamic diagnostic tool, is an appealing concept [4]. Potential targets are circulating tumor cells (CTCs), cell-free DNA (cfDNA) which contains circulating tumor DNA (ctDNA), microRNA signatures, extracellular vesicles, tumor-educated platelets, proteins, and metabolites [5]. Clinical utility has been demonstrated for three cfDNA-based tests that are approved by the U.S. Food and Drug Administration (FDA): the cobas *EGFR* Mutation Test v2 [6], the Epi proColon test [7], and the therascreen *PIK3CA* RGQ PCR kit [8]. Furthermore, the CellSearch system has been approved by the FDA for detection of CTCs in patients with metastatic breast, prostate, and colorectal cancer.

CTCs are shed into the blood by tumor tissue and are commonly considered as precursor cells for metastasis formation [4]. Elevated CTC counts correlate with shortened progression free survival (PFS) and overall survival (OS) in metastatic breast cancer and other metastatic cancers [9]. The CTC count is also a prognostic factor in non-metastatic breast cancer and other non-metastatic cancers [10]. Furthermore, CTC analysis allows the monitoring of treatment response [11].

The predictive utility of CTCs is currently investigated. Whereas some trials such as the SWOG S0500 trial question a clinical utility [12,13,14], others point towards their clinical utility: In the STIC CTC trial first-line treatment of estrogen receptor (ER)-positive metastatic breast cancer patients, either with hormone therapy or chemotherapy, was determined by clinicians or by the baseline CTC level. Patients whose treatment was escalated to chemotherapy based on CTC count had a significantly longer PFS and showed a trend towards a longer OS. Patients whose therapy was deescalated based on a low CTC count had no worse outcome [15].

Here, we present a proof-of-concept study that explores the mutation status combined with further multiparametric characterizations of CTCs to provide additional CTC-based tailored recommendations in clinical practice based on the idea that an in-depth analysis will be highly informative about the evolution of the tumor and lead to an understanding of cancer biology in general and CTC biology in particular. First, whole-exome sequencing (WES) was performed on CTCs from five metastatic breast cancer patients. Next, one patient whose CTCs harbored a mutation targetable with drugs approved by the FDA and the European Medicines Agency (EMA) was selected and the mutation-based treatment recommendation was validated with a detailed multiparametric liquid biopsy analysis over a time period of more than three years. The information gained on the disease and its evolution was used to choose an effective targeted therapy, thus highlighting the potential offered by liquid biopsies.

## 2. Materials and Methods

### 2.1. Patients

Five patients with high CTC counts were selected from the Augusta study (approved by the Ethics Committee of the Medical Faculty of the Heinrich Heine University Düsseldorf; Ref-No: 3430) and DETECT III study (NCT01619111; https://clinicaltrials.gov/ct2/show/NCT01619111 accessed on 26 November 2021). All patients provided their informed consent for the use of their blood samples for CTC analysis and for translational research projects. Clinical patient data are shown in Table 1.

By whole exome sequencing of the CTCs, mutations targetable with drugs approved by the FDA were detected in four patients. From those patients, one was selected to further validate the treatment recommendation by a detailed multiparametric liquid biopsy analysis over a period of more than three years.

The Caucasian 65-year-old female patient 1 was first diagnosed with a multicentric right-sided breast cancer. The breast cancer was ER as well as progesterone receptor (PR) positive and negative for HER2. Staging revealed absence of metastatic lesions. The patient was treated with breast conserving therapy and sentinel lymph node biopsy (pT2 pN0 (0/2 sn) G2 R0 L0 V0, ER-positive, PR-positive, HER2-negative) followed by adjuvant local radiotherapy. In addition, she was treated for five years with an aromatase inhibitor (AI; anastrozole). After 7 years, the patient was first diagnosed with bone metastatic lesions. Therefore, she restarted an endocrine therapy with another AI (letrozole) in combination with the RANKL inhibitor denosumab. A stable disease was observed for 18 months, until she first presented to our department with a newly diagnosed vesical metastatic lesion. Histologic examination showed a positive ER and PR status and a negative HER2 status. A subsequently performed CellSearch analysis revealed 5500 CTCs per 7.5 mL blood. The endocrine therapy was switched to the AI exemestane. Because of osteonecrosis of the jaw, denosumab was suspended. Two months later, CTC numbers increased to approximately 50,000 per 7.5 mL blood. In line, a staging computer tomography (CT) scan demonstrated liver metastatic lesions, progression of bone lesions and suspicion of bone marrow infiltration. The endocrine therapy was stopped and a chemotherapy with weekly epirubicin was started. CTC counts decreased to 3–47 CTCs per 7.5 mL blood. Staging CTs revealed a stable disease of vesical and bone lesions. Hepatic lesions were not radiographically visible anymore. After reaching the cumulative overall dose, epirubicin treatment was switched to fulvestrant (SERD), palbociclib (CDK4/6 inhibitor), and denosumab. CTC counts were measured alongside therapy and fluctuated between 25 and 217 CTCs per 7.5 mL blood. After 13 months, CTC analysis revealed a continuous increase in CTC numbers and CT staging diagnosed new cervical metastatic lesions, a progression of the vesical metastatic lesion, ascites as well as new hepatic metastatic lesions. Moreover, a peritoneal carcinomatosis could not be excluded. A biopsy of the cervical lymph node revealed a positive ER, negative PR, and negative HER2 status of the tumor cells. The treatment regime was switched to weekly paclitaxel. However, weekly paclitaxel was stopped after two administrations because of the further increasing CTC count to 6030 and strong adverse side effects (Figure 2; Supplementary Figure S1).

### 2.2. Intervention

Based on the multiparametric characterization of the CTCs, 5 mg/day everolimus was applied in combination with 20 mg/day tamoxifen and denosumab following clinical guidelines [16].

Primary parameter for monitoring CTC-based therapy were the CTC numbers in peripheral blood, monthly determined by the FDA-approved CellSearch system. In addition, clinical response was measured by computer tomography/magnetic resonance imaging 2.5 months after intervention.

### 2.3. Enrichment and Enumeration of CTCs

CTCs were enriched from peripheral blood or DLA product. DLA was performed as previously described [17,18].

Enrichment and enumeration of CTCs was performed using the CellSearch Circulating Epithelial Cell Kit (Menarini, Florence, Italy) according to manufacturer’s instructions. 7.5 mL blood or 2 × 10^8^ white blood cells (WBCs) from DLA product were used for enumeration of CTCs. The expression of ERα (antibody clone ER119.3) was determined by the CellSearch CXC Kit (Menarini).

Enrichment of CTCs without subsequent automated immunofluorescent characterization was performed using the CellSearch Epithelial Cell Profile Kit (Menarini) according to the manufacturer’s instructions.

### 2.4. Immunofluorescence Analysis

For immunofluorescence analysis cells were fixed with CellSave reagent, permeabilized with 0.1% Triton X-100 (Merck, Darmstadt, Germany), and stained for nucleic acid (DAPI; F. Hoffmann-La Roche, Basel, Switzerland), CK (clone C11, Alexa Fluor 488 conjugated, Cat#: GTX11212, GeneTex, Irvine, United States or clones C11/AE1/AE3, TRITC conjugated, Cat#: CKALLRMB000S, Aczon, Monte San Pietro, Italy), CD45 (clone 35-ZS, Alexa Fluor 647 conjugated, Cat#: sc-1178 AF647, Santa Cruz Biotechnology, Dallas, TX, United States), and caspase cleaved cytokeratin (M30 Cytodeath, FITC conjugated, Cat#: 10800, VLVbio, Nacka, Sweden), Ki67 (clone D3B5, Cat#: 9129S, Cell Signaling Technology, Danvers, MA, USA), phosphorylated Akt (clone D9E, Cat#: 4060, Cell Signaling Technology), or phosphorylated mTOR (clone D9C2, Cat#5536, Cell Signaling Technology). For Ki67 analysis a goat anti-rabbit IgG antibody (Cat# A-11012, Thermo Fisher Scientific, Waltham, MA, USA), for phosphorylated Akt and mTOR a donkey anti-rabbit IgG antibody (Cat# A-21206, Thermo Fisher Scientific) was used as the secondary antibody.

### 2.5. CTC Isolation

Single CTCs were isolated from CellSearch cartridge mainly by micromanipulation with the CellCelector (ALS, Jena, Germany) [19]. For isolation of ERα positive and negative CTCs the DEParray (Menarini) was used.

Larger numbers of CTCs were obtained by FACS sorting (MoFlo XDP sorter, Beckman Coulter, Brea, CA, USA) from DLA product. CTCs were identified by staining for EpCAM (clone VU1D9, Alexa Fluor 488 conjugated, Cat#: 5488S, Cell Signaling Technology), CD45 (clone 35-ZS) and Hoechst 33342 (Thermo Fisher Scientific).

### 2.6. Whole-Genome Amplification

Chromosomal DNA of single isolated cells was amplified by whole genome amplification (WGA) with the Ampli1 WGA Kit (Menarini). Afterwards DNA integrity was determined with the Ampli1 QC Kit (Menarini).

### 2.7. Whole-Exome Sequencing

DNA was purified from FFPE tissue with the GeneRead DNA FFPE Kit (Qiagen, Venlo, The Netherlands). Furthermore, tumor content was determined by staining with hämalaun/eosin and immunohistochemistry. Slides were analyzed by experienced pathologists.

DNA from FACS sorted CTC pellets was extracted using QIAamp DNA Micro Kit (Qiagen).

WES of sorted CTCs (patient 1) or pooled WGA products from CTCs (patients 2, 3, 4, and 5) and matched WBCs was performed using the Agilent Sure Select XT Kit V7 (Agilent, Santa Clara, CA, USA) (patient 1), V6 (Agilent) (patients 4 and 5), or the Illumina IDT Exome Analysis Kit (Illumina, San Diego, CA, USA) (patients 2 and 3). Matched PTs were sequenced using the Agilent Sure Select XT V7 (patient 1) or the Illumina IDT Exome Analysis Kit (patient 3). The integrity of extracted DNA from PTs of patients 2, 4, and 5 was not sufficient to perform WES or no tissue was available. The libraries were sequenced on either a HiSeq 3000 system (Illumina) or a NovaSeq 6000 system (Illumina).

After sequencing, data were uploaded to the Molecular Health Guide platform (Molecular Health, Heidelberg, Germany) and identified single nucleotide variants were analyzed. Variants exceeding a variant allele frequency (VAF) of 5% and exceeding a coverage of 100× with a coverage of that position exceeding 40× in the reference sample were considered reliable.

### 2.8. Clonal Reconstruction

The PT, CTCs from time point 3 (T3), and tumor cells from the bladder metastasis and lymph node metastasis were considered for this analysis. Regions of copy-number neutrality common to all of these samples were determined on the basis of WES copy number profiles obtained with CopywriteR [20] and B-allele frequency analysis. Somatic mutations falling in these regions were selected for analysis with Cloe [21]. Mutations were first clustered with Cloe’s CRP, and Cloe run on the resulting 30 meta-mutations with 6 to 20 clones. Results that achieved the highest log-likelihood were reported.

### 2.9. Sequencing of ESR1 and AKT1 from Single CTCs

ESR1 mutation analysis was performed as previously described [22]. The *AKT1* E17K mutation was analyzed by Sanger sequencing. The amplification was performed by semi nested PCR. Used primers are shown in Supplementary Table S4.

### 2.10. Sequencing of PIK3CA Hotspots by ddPCR

Droplet digital PCR of PTs and single isolated cells was performed by using a QX200™ Droplet Digital PCR system (Bio-Rad Laboratories, Hercules, CA, USA). Primers and probes are shown in Supplementary Tables S5 and S6. For droplet generation the QX200 droplet generator (Bio-Rad Laboratories) was used. Samples were subjected into the Droplet Reader (Bio-Rad Laboratories). Data were analyzed using QuantaSoft analysis software (Bio-Rad Laboratories). MCF7 and T-47D cells were used as a negative and positive control to gate the threshold for finding mutation. Negative controls with no DNA were included at each run.

### 2.11. Array Comparative Genome Hybridization

WGA products were processed for aCGH as previously described [23]; 1 μg DNA was processed for aCGH. As reference, the WGA product of a single GM14667 cell or DNA from matched normal tissue was used. For data analysis, the output image files were normalized and fluorescence ratios for each probe were determined using Feature Extraction software (Agilent; version 10.7.3.1, Protocol CGH_1105_Oct09). Data were visualized and analyzed with the Genomic Workbench 6.5.0.18 software by applying the ADM-2 algorithm with a threshold of 6.0. The centralization algorithm was set to a threshold of 4.0 with a bin size of 10. To identify copy number alterations, an aberration filter with a minimum log_2_ ratio of ±0.3 and a minimum of 100 consecutive probes was set.

### 2.12. FISH Analysis

CTCs enriched by CellSearch were spun on slides and detected using a pan cytokeratin antibody (Cat#: 1835, Biotium, Fremont, CA, USA). As secondary antibody, a donkey anti-rabbit IgG antibody (Cat# A-31573, Thermo Fisher Scientific) was used. Afterwards slides were pretreated with SSC Wash Buffer (Zytovision, Bremerhaven, Germany), pepsin (Zytovision), and formaldehyde (Merck, Darmstadt, Germany). DNA was then denatured and ZytoLight SPEC ESR1/CEN 6 Dual Color Probes (Zytovision) were hybridized overnight at 37 °C. For washing Wash Buffer A (Zytovision) was used.

### 2.13. Multiplex ESR1-ddPCR from cfDNA and Data Analysis

For screening of *ESR1* mutations plasma was collected from EDTA vacutainers (Becton, Dickinson and Company, Franklin Lakes, NJ, USA) immediately after blood draw and cryo-conserved. The cfDNA was extracted from 2 mL plasma using the QIAamp Circulating Nucleic Acid Kit (Qiagen). Screening for *ESR1* mutations was performed as previously described by using a multiplex ddPCR [24]. Samples were considered positive if the merged replicates presented a minimum of 3 E380Q mutant droplets or 8 exon 8 mutant droplets and if the average mutant allele frequency was higher than 0.1%.

### 2.14. RNA Sequencing

A total of 40,000 CTCs were sorted from DLA product fixed with 4% paraformaldehyde. RNA sequencing was performed in duplicates using the TruSeq RNA Access Library Kit (Illumina). Libraries were sequenced on a NextSeq 500 system (Illumina). Data were analyzed and TPM values were determined using the CLC Genomics Workbench (Qiagen). Reads were mapped by applying the following parameters: mismatch cost: 2; insertion/deletion cost: 3; length fraction: 0.8; similarity fraction: 0.8; global alignment: no; strand specific: both; maximum number of hits per read: 5.

### 2.15. CTC Culture

Viable CTCs were enriched from a cryo-conserved DLA product by using the Parsortix system and were cultured as previously reported [25]. Briefly, CTCs were cultured in low attachment plates (Corning, Corning, NY, USA) with RPMI 1640 medium supplemented with 1 × B27 (Thermo Fisher Scientific), 20 ng/mL human epidermal growth factor (Merck), 20 ng/mL fibroblast growth factor (Merck), and 1% penicillin-streptomycin (Thermo Fisher Scientific) in a humidified atmosphere with 5% CO2 and 4% O2.

For drug testing 100 CTCs were seeded per well of a 96-well plate after ten days of pre-culture. The cells were treated with capivasertib (MedChem Express, Monmouth Junction, NJ, USA), everolimus (Merck), epirubicin (Merck), and paclitaxel (Merck). Each drug concentration was tested in triplicates. After incubation for 6 days, cells were spun on glass slides, stained for cytokeratin, and numbers were determined by counting. As references, cell lines MDA-MB-231, SK-BR-3, T-47D, and MCF7 were used (ATCC, Manassas, VA, USA; catalog numbers: MDA-MB 231: HTB-26, SK-BR-3: HTB-30, T-47D: HTB133, and MCF7: HTB-22). Cells were authenticated via short tandem repeat analysis and regularly tested negative for *Mycoplasma*.

### 2.16. Statistical Analysis

Statistical analyses were performed using GraphPad Prism (Graphpad Software, San Diego, CA, USA). *P*-values < 0.05 were considered statistically significant.

## 3. Results

### 3.1. Whole-Exome Sequencing of CTCs to Provide CTC-Based Treatment Recommendations

CTCs from five metastatic breast cancer patients were isolated and analyzed by WES. All patients had a PT of luminal subtype. At the time of CTC mutation analysis, the patients’ CTC counts per 7.5 mL of blood were between 94 and approximately 50,000 (Table 1).

**Figure 1:**
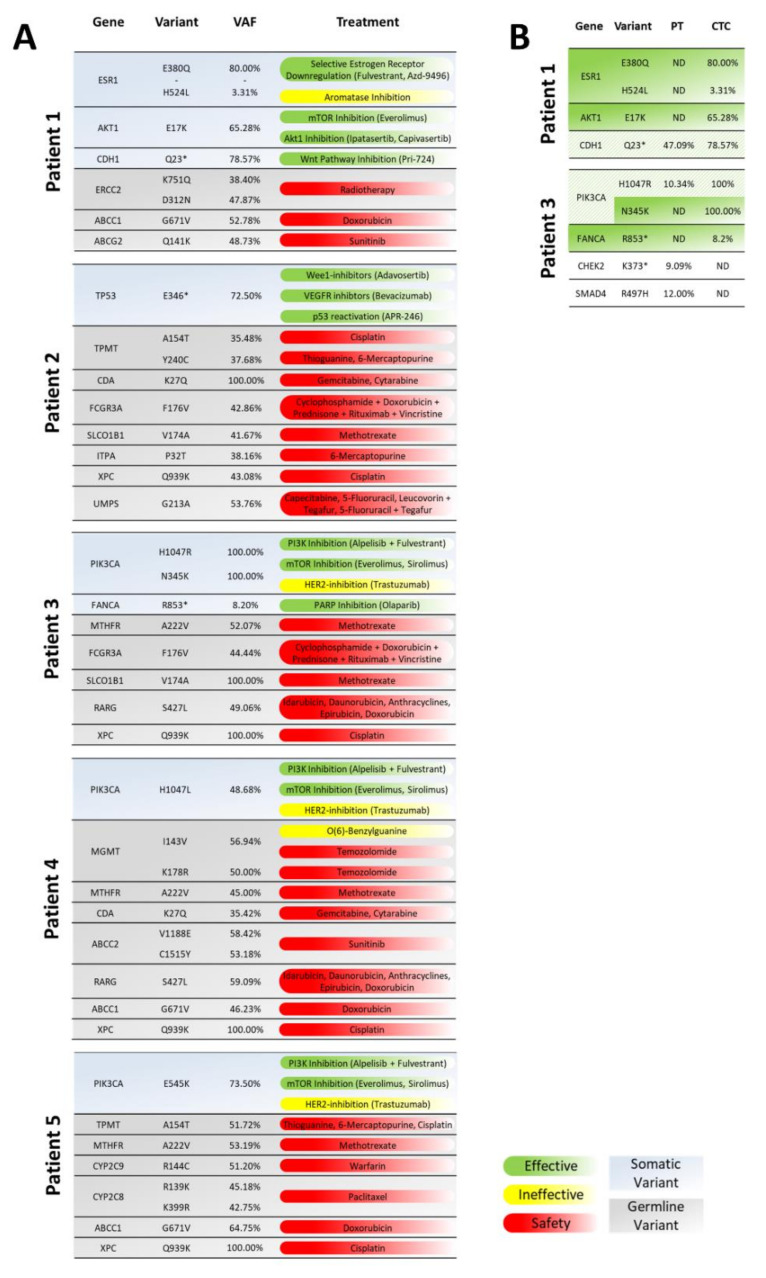
Mutations detected by whole-exome sequencing (WES) of circulating tumor cells (CTCs) and the therapies targeting them: (**A**) Only clinically relevant mutations detected in the CTCs are shown. The second exon of the *AKT1* gene was not covered in the WES of all WGA samples. Because this exon contains amino acid E17, frequently mutated in metastatic breast cancer, it was sequenced also with targeted Sanger sequencing (Supplementary Figure S2). For each detected mutation, treatments and their efficacies and safety concerns are listed (effective: potentially effective treatment option; ineffective: potentially ineffective treatment; safety: potential safety concern). (**B**) Concordance of mutations between primary tumor (PT) and CTCs. Only clinically targetable mutations are listed. Variants that were detected only in CTCs but not in the PT are shown in green. Variants that were detected in a minor subclone of the PT compared to the CTCs are indicated in green hatched. nd (not detected).

Mutations targetable by specific therapies were identified in CTCs from all patients (Figure 1A, Supplementary Figure S2). In patient 1, we identified an *ESR1* E380Q mutations with a VAF of 80.0% and an *AKT1* E17K mutation with a VAF of 65.3%. The *ESR1* E380Q mutation leads to a conformational change in the protein and to a ligand-independent ER activation, thereby conferring resistance to aromatase inhibition [26]. Despite this, second-generation selective estrogen receptor degrader such as fulvestrant retain activity [27]. The *AKT1* E17K mutation hyperactivates the mTOR pathway. This pathway could be targeted by mTOR inhibitors like everolimus or AKT-inhibitors like ipatasertib and capivasertib [28,29]. Furthermore, a *CDH1* Q23* mutation was observed in both the PT with a VAF of 47.1% and in CTCs with a VAF of 78.6%. CDH1 is a negative regulator of beta-catenin and inactivates the canonical WNT signaling pathway which normally inhibits cell proliferation and differentiation [30]. A nonsense mutation might lead to an activation of WNT signaling and therefore might be targeted with WNT-pathway inhibitors, which have shown efficacy in pre-clinical and clinical trials in multiple cancer types [31].

The analysis of the CTCs from patient 2 revealed a mutation in the tumor suppressor gene *TP53*. Nonsense mutations in *TP53* are likely to confer loss of its tumor suppressor activity [32]. Preclinical and clinical data suggest that the Wee1-inhibitor adavosertib may increase sensitivity towards DNA-damaging agents in tumors with loss of *TP53* [33]. In clinical studies antiangiogenic agents targeting VEGF/VEGFR signaling such as bevacizumab and pazopanib improved the outcome of patients with *TP53* mutations [34,35]. Furthermore, preclinical and initial clinical data showed that the p53-reactivating drug APR-246 may be effective in *TP53*-mutated cancers and may have synergistic or additive effects with other anti-cancer agents, such as chemotherapy [36].

Analysis of the CTCs from patients 3, 4, and 5 revealed the presence of *PIK3CA* mutations. The *PIK3CA* gene encodes for the p110α subunit of the phosphoinositide-3-kinase (PI3K) which promotes cell proliferation and survival by activation of the PI3K/AKT signaling pathway [37]. Patient 3 harbored the mutations H1047R and N345K with VAFs of 100%, patient 4 had a H1047L mutation at a VAF of 48%, and in patient 5 the mutation E545K was detected with a VAF of 72%. The detected variants strongly activate the downstream pathway [38] and offer the chance to be targeted by PI3K inhibitors. For the treatment of hormone receptor-positive, HER2-negative advanced breast cancers harboring such variants, alpelisib plus fulvestrant is indicated [39]. HER2-positive tumors of patients owing this variant showed resistance to trastuzumab [40]. Additionally, preclinical models with this variant are sensitive to the mTOR inhibitors everolimus and sirolimus [41].

Furthermore, the CTCs from patient 3 harbored a nonsense mutation in the *FANCA* gene. FANCA is involved in DNA damage repair and in the maintenance of genomic stability. Nonsense mutations in FANCA are likely to disrupt function and lead to deficiencies in DNA repair [42]. In preclinical studies, loss of functional FANCA sensitized cells to PARP inhibitors such as olaparib [43].

In addition to the CTC analysis, mutational analyses of the PTs were performed by WES for the patients 1 and 3 and ddPCR on the mutated *PIK3CA* positions for the patients 4 and 5. The clinically targetable mutations of *ESR1* and *AKT1* were not detected in the PT of patient 1. In the PT of patient 3, the *PIK3CA* mutation H1047R was present in only a minor part of the cells as indicated by a VAF of 13%, whereas the *PIK3CA* mutation N345K was not detected (Figure 1B; Supplementary Figure S3).

In time matched needle biopsies of patients 4 and 5, the mutational status of the *PIK3CA* gene was investigated by ddPCR. In both cases, *PIK3CA* mutations similar to those in the CTCs were detected (Supplementary Table S1).

### 3.2. Analysis of Mutations and Chromosomal Aberrations during the Course of the Disease

To validate the above findings and eventually treat the patient accordingly, we performed a detailed longitudinal and multiparametric analysis on the CTCs of patient 1. CTC based treatments were not considered for the other patients because, first, the clinical situation of some of the patients worsened quickly or, second, the PI3K inhibitor alpelisib was not yet approved by the EMA.

**Figure 2:**
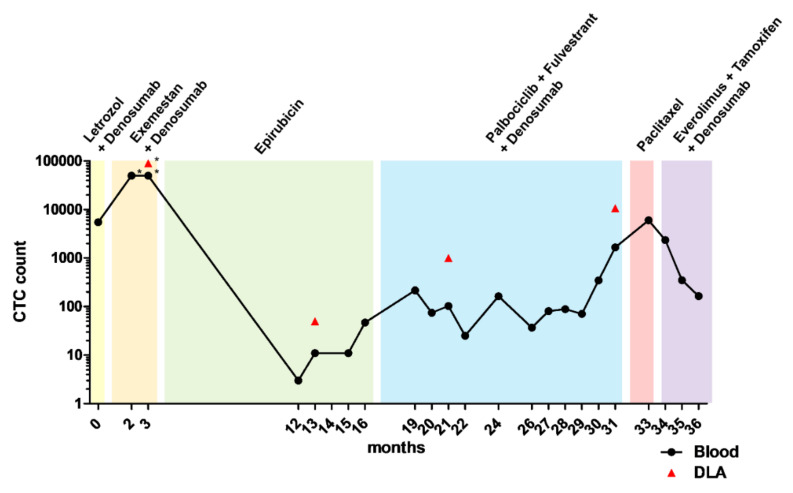
Circulating tumor cell (CTC) count dynamics of patient 1 during the course of the disease. CTCs from patient 1 were counted from 7.5 mL blood or 2 mL diagnostic leukapheresis product (DLA) using the CellSearch system. Each color indicates the duration of a different line of therapy. Time point T0 corresponds to the first presentation of the patient to our department, approximately seven years after the resection of the primary tumor. *The CTC count exceeded the CellSearch detection range. CTC count was estimated by dilution.

**Figure 3:**
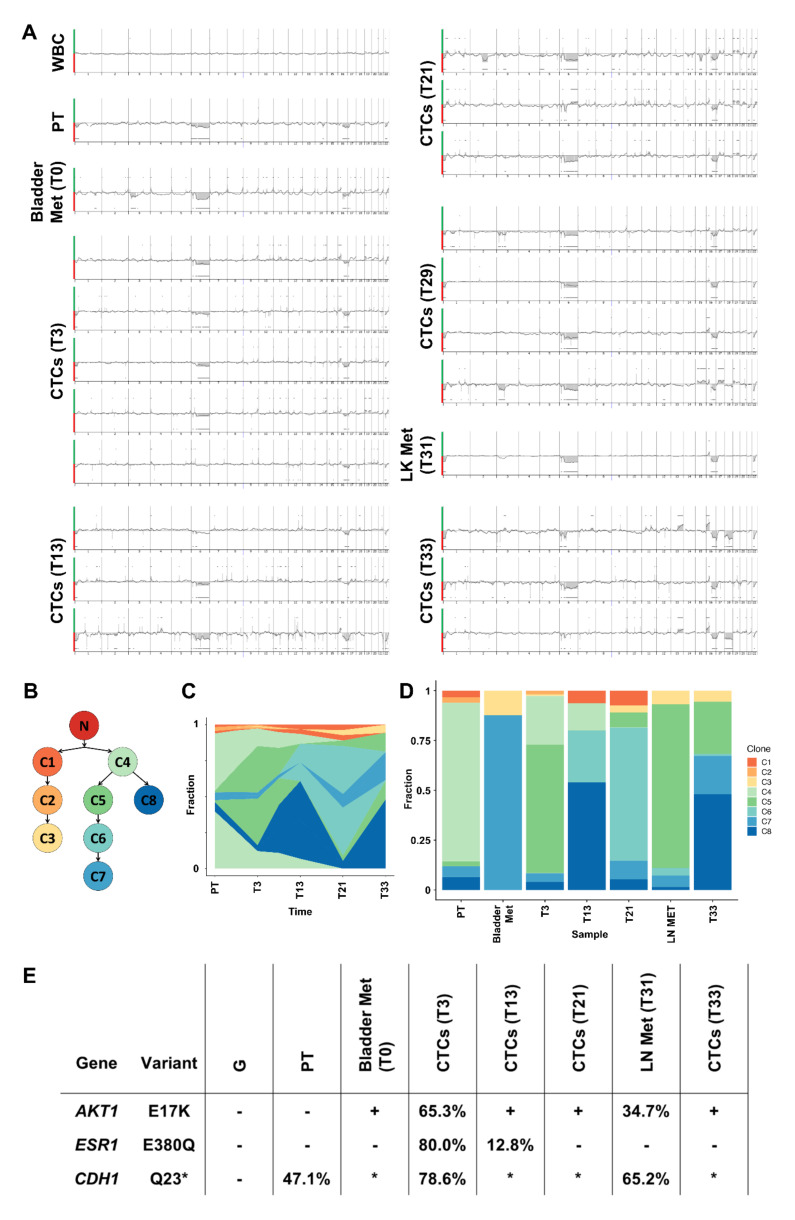
Clonal evolution of the tumor of patient 1: (**A**) Chromosomal aberrations as determined by array comparative genome hybridization. Green indicates a signal above the baseline representing a copy number gain. Red indicates a signal below the baseline representing a copy number loss. (**B**) Clonal reconstruction from the whole-exome sequencing data revealed eight tumor clones. (**C**,**D**) Clonal dynamics of the tumor cell clones during the course of the disease. (**E**) Presence and dynamics of clinically relevant mutations. Values indicate variant allele frequencies. Positive results (no allele fraction) come from Sanger sequencing (Supplementary Table S2). * The *CDH1* Q23 position was not covered by whole-exome sequencing on amplified DNA samples. No information about the mutational status in those samples is available. CTC (circulating tumor cell); WBC (white blood cell); PT (primary tumor); LN (lymph node); Met (metastasis); G (germline).

During the course of treatment mutations and chromosomal gains and losses were analyzed comprehensively in the PT, the bladder metastasis from T0, the cervical lymph node metastasis from T33, as well as single CTCs from T3, T13, T21, T29, and T33 (Figure 2, Figure 3A, and Supplementary Figure S4). According to array comparative genome hybridization (aCGH), the PT showed losses of parts of chromosome 1p, of a large part of chromosome 6, and of parts of chromosomes 16q, 17p, and 22. This profile overlapped with the whole exome sequencing data. The bladder metastasis and lymph node metastasis had an aberration profile similar to that of the PT, although the lymph node metastasis was detected and sampled 31 months after the bladder metastasis. An additional loss of chromosome 3p was detected in both metastases. CTCs at T3 exhibited largely the same aberrations as the PT and these aberrations were widely congruous. The genetic profiles of CTCs from T13 and from the later time points T21 and T29 were largely consistent with the profiles detected in CTCs isolated at earlier time points. However, individual CTCs at T29 also exhibited a loss on chromosome 3p as seen in the bladder metastasis and lymph node metastasis. At T33, this chromosomal loss was no longer observed while gains on chromosomes 13q and 16q and a loss of chromosome 18 were acquired. However, in some CTCs, at least within the applied detection thresholds, the partial loss of chromosome 6 disappeared, which was so far present in almost all analyzed CTCs and tissue samples: instead, these CTCs were characterized by only a minor loss on chromosome 6p. Furthermore, a loss in the X chromosome was identified in CTCs collected at T33. The aberrations that were detected at the single-cell level were also confirmed by WES of pooled WGA products (Supplementary Figure S4).

The evolution of the tumor cells was further investigated by clonal reconstruction, which highlighted the dynamics of different tumor clones during the course of the disease. Eight clones were identified based on mutations and placed in a branched phylogeny. The majority of mutations was found at low levels in all samples (Supplementary Figure S5). These mutations modelled with clones on the left branch (tumor clones 1–3) (Figure 3B; Supplementary Figure S6). The PT sample is mainly composed of tumor clone 4, the progenitor of clones on the right branch (tumor clones 5–8). Subsequent samples—the bladder metastasis, the lymph node metastasis, and all CTC samples—showed an expansion of these clones that are descendants of clone 4. At T13 the majority of the CTCs stemmed from tumor clone 8. This remained a minority tumor clone in blood samples taken at the following time points, until it became dominant again in the CTCs when the tumor acquired resistance to palbociclib/fulvestrant and later on did not respond to paclitaxel (Figure 3C,D).

Next, the presence of clinically relevant mutations during the course of the disease was analyzed. Unlike the PT, where no clinically relevant mutations were detected by WES, DNA analysis of the bladder and lymph node metastases showed the activating *AKT1* mutation E17K. Similarly, this *AKT1* mutation was present in CTCs ever since (Supplementary Table S2). Moreover, CTCs from T3 harbored the *ESR1* mutation E380Q with a VAF of 80.0%. The VAF of this mutation reduced to 12.8% at T13, and was undetectable in CTCs isolated at later time points (Figure 3E).

**Figure 4:**
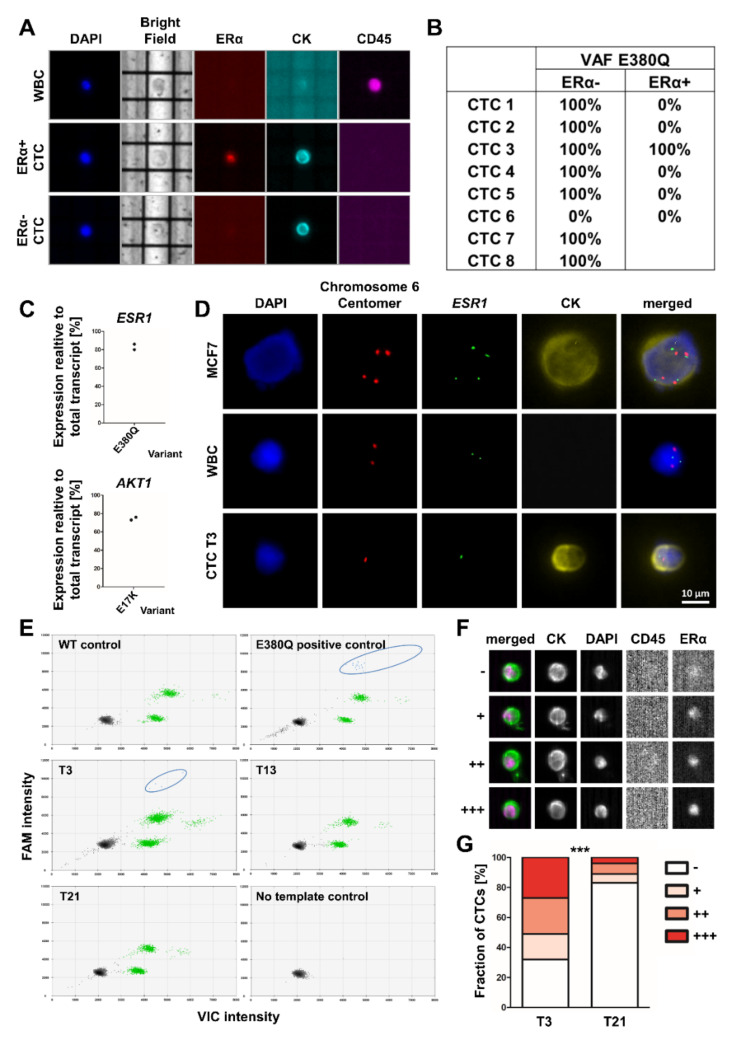
Analysis of *ESR1* mutations and ERα expression during the course of the disease of patient 1. (**A**,**B**) The *ESR1* gene from single ERα positive or negative circulating tumor cells (CTCs) from T3 was sequenced (images were generated with the DEParray system; original magnification 10×). (**C**) Frequencies of the variant allele on transcriptome level were determined by RNA sequencing of the primary tumor and CTCs from T3 (2 replicates each). (**D**) *ESR1* copy numbers determined by fluorescence in situ hybridization. Signals detected with a probe for the *ESR1* gene are shown in green. As a reference, a probe for the centromere region of chromosome 6 was used (red). Tumor cells were identified by cytokeratin positivity (original magnification 40×). (**E**) ddPCR analysis of *ESR1* E380Q mutation in ctDNA. Droplets harboring a mutation are highlighted with circles (blue). (**F**,**G**) ERα expression analyzed with the CellSearch system. CTCs were classified into four groups based on the ERα staining intensity (images were generated with the CellSearch system; original magnification 10×). The statistical significance level was determined by a Fisher’s exact test by comparing ERα-positive (+/++/+++) and ERα-negative CTCs (*** *p*-value < 0.001). WBC (white blood cell); WT (wildtype).

Both, *ESR1*-mutant and *ESR1*-wildtype CTCs could be ERα-positive or ERα-negative (Figure 4A,B). The observed *AKT1* and *ESR1* mutations were also detected at the transcriptomic level. The *ESR1* mutation E380Q was observed with a frequency of 83%. The *AKT1* E17K transcript showed a frequency of 74.5% (Figure 4C). In line with the aberrations detected in chromosome 6 in CTCs from T3, FISH analysis confirmed the absence of one *ESR1* allele and one centromere of chromosome 6 (Figure 4D).

To analyze the lack of detection of *ESR1* mutations on CTCs at later points of the course of the disease further, an analysis of *ESR1* hotspot mutations at positions E380, Y537 and D538 was performed on ctDNA. At T3, the E380Q mutation was identified with a frequency of 0.2%. In concordance with the CTC analysis, no *ESR1* mutations were detected at T21. However, *ESR1* mutations could not be detected in the ctDNA at T13, contrary to the CTC results (Figure 4E). Mutations at the positions Y537 and D538 were not detected at any time point (Supplementary Table S3).

In addition, the proportion of ERα-positive CTCs was investigated. At T3, 68% of the CTCs showed a nucleated ERα staining. At T21, during therapy with palbociclib and fulvestrant, the proportion of ERα-positive cells dropped to 17% (Figure 4F,G).

### 3.3. CTCs Exhibit No Indications for Proliferation but Show Reduced Apoptosis

**Figure 5:**
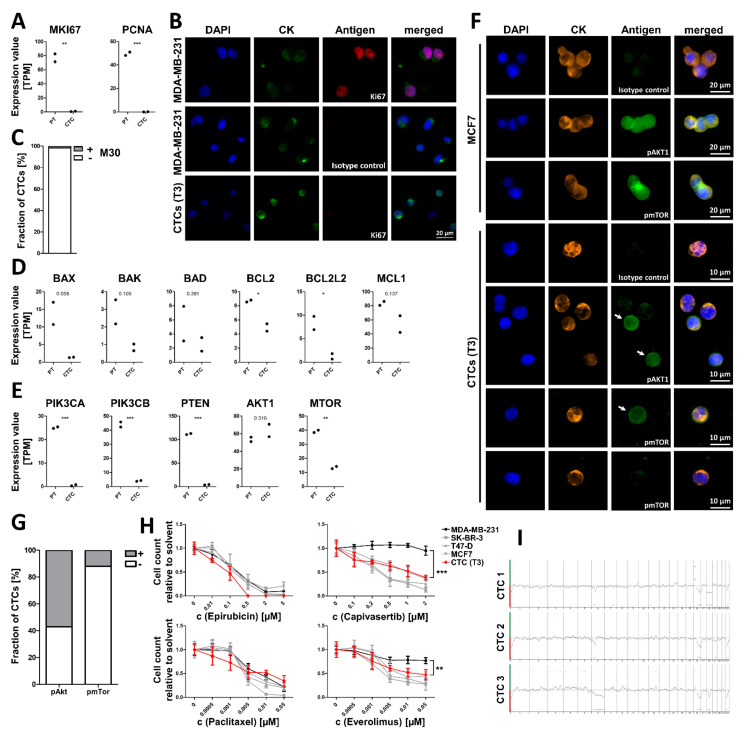
Analysis to predict the efficacy of a treatment targeting the AKT1/mTOR pathway in patient 1: Gene expression data were generated by RNA sequencing of bulk circulating tumor cells (CTCs) and the primary tumor (2 replicates each). Statistical significance levels were determined with a two-tailed *t*-test. Selected genes are involved in proliferation (**A**), regulation of apoptosis (**D**), and regulation of the PI3K/AKT1/mTOR pathway (**E**). (**B**) Expression of the proliferation marker Ki67 analyzed by immunocytofluorescence analysis. Ki67 positive cells from the MDA-MB-231 cell line served as references (original magnification 40×). (**C**) Apoptosis status as determined by staining for caspase cleaved cytokeratin 18 (M30) (Supplementary Figure S7). Positive cells were deemed apoptotic. (**F**) Immunofluorescence analysis of phosphorylated AKT1 (Ser473) and phosphorylated mTOR (Ser2448). Cells from the MCF7 cell line served as positive control. Positive CTCs are highlighted with arrows (original magnification 40×). (**G**) Ratio of CTCs positive/negative for phosphorylated AKT1 and phosphorylated mTOR. (**H**) The drugs epirubicin, capivasertib, paclitaxel, and everolimus were tested with in vitro cultured CTCs. As references, cells from SK-BR-3, T47-D, MCF7 (all sensitive to treatment with capivasertib and everolimus; shown in grey), and MDA-MB-231 cell lines (resistant to treatment with capivasertib and everolimus; shown in black) were used. Statistical significance levels were determined by a two-way ANOVA with post hoc Bonferroni test. Error bars show standard deviation. (**I**) Copy number aberration profiles of remaining CTCs at T36. Green indicates a signal below the baseline representing a copy number gain. Red indicates a signal below the baseline representing a copy number loss. *** Indicates a *p*-value < 0.001, ** indicates a *p*-value < 0.01, * indicates a *p*-value < 0.05; TPM (transcripts per million); CK (cytokeratins).

Patients with several thousand CTCs per 7.5 mL blood are extremely rare. To investigate the reason behind such high numbers, we analyzed the expression of markers related to proliferation and apoptosis in the PT and CTCs from T3. In comparison to tumor cells from the PT, in CTCs the expression levels of *PCNA* and MKI67 was reduced 495.5-fold (*p*-value 0.0008, two-tailed *t*-test) and 81.9-fold (*p*-value 0.0054, two-tailed *t*-test), respectively (Figure 5A). The Absence of Ki67 in CTCs was confirmed by immunofluorescence analysis (Figure 5B).

### 3.4. In Vitro treatment of Cultured CTCs

Based on the mutation analysis and the expression analysis, AKT1 was deemed a disease driver in this patient. Therefore, AKT1 or mTOR inhibitors were considered as potential drugs. The efficacy of the AKT1 inhibitor capivasertib and the mTOR inhibitor everolimus were tested on CTCs in vitro and compared to the cytostatic drugs epirubicin and paclitaxel using cultured CTCs from a diagnostic leukapheresis (DLA) product obtained at T3. As references the triple-negative cell line MDA-MB-231, the ERα-positive and *PIK3CA*-mutated cell lines T-47D and MCF7, and the *ERBB2*-amplified cell line SK-BR-3 were used. All cell lines perished in the presence of epirubicin and paclitaxel, but only T-47D, MCF7, and SK-BR-3 responded to capivasertib and everolimus. For drug testing, CTCs from T3 were short-term cultured. These CTCs were highly sensitive to epirubicin treatment validating the clinicians’ decision for treatment at this time point. Paclitaxel was as effective as in the reference cell lines. Furthermore, the CTCs responded to the treatment with capivasertib and everolimus targeting the AKT1/mTOR pathway (Figure 5H).

### 3.5. CTC-Based Treatment

Based on the above findings, treatment of the patient was switched after paclitaxel to the mTOR-inhibitor everolimus, combined with the selective estrogen receptor modulator tamoxifen according to current therapeutic guidelines. Within 17.5 weeks, the CTC count dropped by 97.3% from 6030 CTC to 165 CTC per 7.5 of blood (Figure 2). Staging 2.5 months after treatment with everolimus plus tamoxifen and denosumab showed a stable disease situation of the metastatic lesions. The chromosomal aberrations detected on the remaining CTCs at time point T36 were largely similar to those detected on CTCs at T33 (Figure 5I). The *AKT1* E17K mutation was also still detected in the majority of these CTCs (Supplementary Table S2).

## 4. Discussion

CTCs are considered as a prognostic marker in metastatic breast cancer. However, their clinical utility is still under investigation and has not been conclusively demonstrated. Here, we tested their clinical utility in a proof-of-concept study starting with CTC WES from five metastatic luminal breast cancer patients.

Therefore, the CTCs were enriched from blood or DLA product with the FDA approved CellSearch system. Single CTCs were isolated, their DNA was amplified and high-quality WGA products were pooled for WES. By this approach, mutations that can be addressed by FDA approved therapies were detected in the *PIK3CA*, the *ESR1,* and the *AKT1* genes in four patients, including mutations that were not detected in the PT. Tumor cells with *PIK3CA* mutations could be targeted by the PI3K inhibitor alpelisib plus fulvestrant in ERα positive tumors. Mutations leading to a constitutive activity of the ERα convey a resistance to aromatase inhibition. Tumor cells with an activating *AKT1* mutation can be targeted by AKT1 inhibitors or agents blocking mTOR, a downstream member of the AKT1 signaling pathway. The tailored therapies were recommended to the clinicians and turned out to be especially clinically relevant for one patient.

In the following, a longitudinal and multiparametric liquid biopsy analysis of one index patient was performed to track down the developmental history of the tumor. Clustering analysis of mutations that were identified by WES led to the identification of eight tumor cell clones belonging to two major branches. While it looks like that there are two independent origins of the tumor, the most likely explanation is that the mutations common to both branches were not included in the analysis because they were not called, filtered out or have been affected by copy number alterations. Nearly all tumor clones were already present in the PT. Furthermore, in tissue from both analyzed metastases, several different tumor cell clones have been detected. This suggests the seeding of multiple tumor cell clones to the metastatic niche, either by co-seeding of multiple single tumor cells [44] or by seeding of tumor cell clusters consisting of tumor cells of multiple clones, which might have survival advantages and an enhanced metastatic potential compared to single CTCs [45]. Indeed, some CTC clusters were observed by CellSearch analysis at most time points. Of note, the bladder metastasis and lymph node metastasis shared very similar copy number aberration profiles leading to the assumption of an early seeding event. As the metastases, the CTC populations consisted of different tumor cell clones at all time points. This heterogeneity is underlined by the aCGH analyses of single CTCs, also showing different copy number aberration profiles especially at T21 and T33. Furthermore, a highly dynamic evolution of the tumor was observed: The *ESR1* mutation E380Q was identified at T3 and T13. This mutation was absent in the tissue biopsies and no CTCs with this mutation were found upon epirubicin treatment. We assume that the *ESR1* mutant subclones that developed during estrogen deprivation were widely eradicated by the chemotherapy with epirubicin. During the epirubicin therapy, the majority of the analyzed CTCs belonged to the tumor clone eight. This observation leads to the assumption that this clone was especially resistant towards chemotherapy. During therapy with palbociclib and fulvestrant clone 8 became less abundant. However, at T31 most of the analyzed CTCs belonged to the tumor cell clone 8 and were later resistant towards a chemotherapy with paclitaxel.

Our analysis identified the *AKT1* E17K mutation as the putative driver of the tumor. This mutation was first detected in a biopsy of the bladder metastasis and found in the majority of the CTCs ever since, as well as in a lymph node metastasis biopsied at T31. AKT1 is a member of the serine-threonine kinase class and plays a key role in cellular processes, including growth, proliferation, survival, and angiogenesis. The E17K mutation leads to a pathologic association of *AKT1* with the plasma membrane and constitutive activation of the protein which, in turn, results in an increased level of AKT1 phosphorylation and activation of downstream molecules independent of upstream events, e.g., stimulation by growth factors [46]. For breast cancer patients, *AKT1* E17K mutation frequencies between 1.4% and 8.2% have been described in tissue [47].

Based on our findings, a therapy with the mTOR inhibitor everolimus in combination with tamoxifen was recommended to the patient when a progress of the tumor was observed under therapy with palbociclib and fulvestrant at T31. The patient initially rejected it and preferred a chemotherapy with paclitaxel. However, during the treatment with paclitaxel the clinical situation worsened, the CTC numbers dramatically increased and the patient was suffering from severe side effects. Thus, the patient agreed to the liquid biopsy-based therapy eventually. Based on the detection of this mutation and the following characterization of the CTCs, the patient was treated with the mTOR inhibitor everolimus. Alternatively, patients with activating *AKT1* mutations could be treated with specific AKT1 inhibitors. Such drugs like capivasertib are currently tested in clinical trials. The efficacy of capivasertib on luminal breast cancer has been shown in combination with paclitaxel in the BEECH trial and in combination with fulvestrant in the FAKTION trial [48,49]. Especially patients with an *AKT1* mutated tumor could benefit from AKT1 inhibition by capivasertib [28]. However, AKT1 inhibitors have not yet been approved for the treatment of breast cancers. Here, we could show that the CTCs harboring the *AKT1* E17K mutation respond to capivasertib in vitro. The patient responded well to the liquid biopsy-based therapy and a reduction in the CellSearch determined CTC count by 97.3% was observed during a time period of 17.5 weeks, in which no progression of the metastasis was detected. The chromosomal aberrations uncovered on CTCs by aCGH did not differ from those before the everolimus/tamoxifen therapy leading to the assumption that within that observation period no new and potentially resistant tumor clone had occurred.

The efficacy of a treatment, which is based on a liquid biopsy mutation analysis, is currently being investigated in several clinical trials: In the PADA-1 trial *ESR1* mutated patients are treated with CDK4/6 inhibition in combination with fulvestrant [50]. In the multiple parallel cohort trial plasmaMATCH targetable mutations in several genes are identified and patients are treated accordingly [51]. The SOLAR-1 trial is analyzing the efficacy of alpelisib on tumor harboring activating *PIK3CA* mutations and can already show a benefit for such patients, which led to the approval of alpelisib and the therascreen PIK3CA RGQ PCR kit by the FDA [39].

In all the trials mentioned above, mutations are identified by ctDNA analysis. Although digital PCR-based methods have demonstrated to have suitable clinical sensitivity considering that digital PCR and BEAMing can detect somatic point mutations at a sensitivity range of 1% to 0.001%, these technologies require prior knowledge of the region of interest to detect known mutations given the need for the PCR assay to be designed accordingly [1]. Whether the analysis of ctDNA or CTCs is superior has been discussed extensively with the conclusion that both analytes might be complementary [52]. Here, we show the detection of the *ESR1* E380Q mutation in CTCs although no *ESR1* mutations have been detected from ctDNA at the same time. This might be because the analysis of a higher number of CTCs from DLA product enables to identify mutations in minor subclones. Furthermore, the analysis of CTCs offers the potential to detect novel mutations as therapeutic targets. 

However, the analysis of CTCs is challenging due to low CTC numbers and a significant number of cancer patients without detectable CTCs. This limitation could at least partly be solved by our approach of using DLA to pre-enrich MNCs from several liters of the patients’ blood. DLA increases the number of CTC positive patients and the CTC yield per patient [17,18]. Furthermore, its implementation into the clinic is unproblematic and no adverse events are observed [17]. However, the characterization of CTCs at single cell level has so far mainly been limited to proof-of-concept studies and it remains unclear to which extend a limited number of CTCs reflects the heterogeneity of the tumor [53].

To maximize the clinical utility of liquid biopsies, implementation of novel multiparametric strategies to combine information from multiple sources might play a key role [5]. Functional analysis of living CTCs could especially add another dimension to conventional liquid biopsies and can help to deepen the understanding on the CTCs’ biology [54]. As previously described, DLA facilitates the obtaining of high numbers of viable CTCs for culture that can be cryo-conserved for later usage [25]. Although culturing CTCs is still highly challenging, we were successful in enriching living cells from the blood and from the DLA product at T3. Due to the high CTC count at that time, there was no need for a time-consuming pre-culture that could potentially lead to artefacts in the response to the tested drugs by long-term in vitro culture. The observed results from a panel of tested drugs matched with the observations of the clinical situation: The cultured CTCs responded to therapies targeting the *AKT1* E17K mutation and showed a high sensitivity towards epirubicin. However, we also observed the CTCs were sensitive towards paclitaxel, which is contradictious with the clinical observations. We assume this to be the case because the resistance towards a later chemotherapy was mainly provided by CTCs belonging to tumor clone 8. This tumor clone was widely underrepresented at T3 when the CTCs for the drug test were isolated compared to the time when the tumor was treated with paclitaxel, but was selected during epirubicin treatment.

We are aware that our ‘index patient’ may only represent a minority of all breast cancer patients. She had, at least at some time points of blood collection, extraordinary high numbers of CTCs in her blood circulation. We assume that this was due to reduced apoptosis and greater survival mediated by the activation of the Akt1 protein. High CTC numbers simplify their analysis. However, due to recent developments in single cell analysis, clinical relevant information on the genome, transcriptome, or proteome can be gained from single CTCs [55,56]. Mutation data on CTCs from patients 2, 3, 4, and 5 were gained from pooled WGA products from 6 to 15 cells. Mutation analyses of the CTCs from patient 1 at later time points were also generated from WGA products from few single CTCs.

## 5. Conclusions

In this study, we show a workflow for WES of amplified DNA from low CTC numbers to identify clinically relevant mutations, combined with a detailed multiparametric liquid biopsy analysis that can personalize and optimize patient therapy based on liquid biopsies. We propose that in a clinical setting such an analysis should be started with a screening for CTC positive patients. For patients with lower CTC numbers, DLA should be considered. Next, the CTCs DNA should be amplified and analyzed by whole exome sequencing. In case of the detection of mutations, which can be targeted with drugs that are clinically suitable for the particular patient, in a major portion of the tumor cell, we recommend a longitudinal and multiparametric analysis to validate the findings. Such an analysis can exploit the strengths of liquid biopsies to be performed regularly over a period of time without any clinical complications as well as the opportunities of CTCs as a multiparametric analyte, ultimately enabling a drug test if CTCs can be cultured in vitro. If the CTCs respond to the selected drug, the CTC-based therapy can be applied to the patients.

In conclusion, we present here a longitudinal multiparametric liquid biopsy analysis of a breast cancer patient and demonstrated the potential of this approach to identify actionable alterations that can guide and validate individualized treatment decisions in real time, monitor treatment response, and influence clinical outcome. Finally, from a scientific point of view, such studies can provide valuable additional insights into the biology and dynamic response of CTCs to different treatment regimens.

## Figures and Tables

**Table 1:** Clinical data.

Patient ID	Age at CTC Sequencing	Type	M	G	ER	PR	HER2/neu	Chemo-Therapy	Radiation	Endocrine Therapy	CTC Count per 7.5 mL Blood	Cells Used for WES	Material CTCs Were Obtained from for WES
Patient 1	74	Invasive-lob	Bone, bladder *	2	+	+	−	−*	+	+	Approx. 50,000	20,000 CTCs	DLA product
Patient 2	70	Invasive-lob	Bone, liver	2	+	−	−	+	+	+	2687	8 CTCs (WGA)	Blood
Patient 3	64	Invasive-lob	Bone, LN, ovary, pleura	2	+	+	−	+	+	+	583	6 CTCs (WGA)	Blood
Patient 4	72	Invasive-lob	Bone, BM	2	+	+	−	+	+	+	8000	15 CTCs (WGA)	Blood
Patient 5	51	NST	Bone, liver	2	+	+	−	+	+	+	94	9 CTCs (WGA)	Blood

* Clinical data from the beginning of the observation period at T3 when whole exome sequencing (WES) was performed. Later, patient 1 developed metastatic lesions in the liver and lymph nodes and received chemotherapy—first epirubicin, then paclitaxel. ^†^ Determined by CellSearch system M (metastasis sites); G (grading); ER (estrogen receptor); PR (progesterone receptor); lob (lobular); NST (no special type); LN (lymph node); BM (bone marrow); WGA (whole genome amplification); DLA (diagnostic leukapheresis)

## Data Availability

Whole-exome and RNA sequencing raw data can be requested from the corresponding authors.

## Supplementary Materials

The following are available online at https://www.mdpi.com/article/10.3390/cancers13236004/s1, Figure S1: Staging CT analysis of patient 1; Figure S2: Mutation analysis of the *AKT1* E17K hotspot; Figure S3: Whole exome sequencing of the primary tumor of patients 1 and 3; Figure S4: Copy number profiles determined by whole-exome sequencing; Figure S5: Allele fractions of clustered mutations; Figure S6: Genotypes of the identified tumor clones, in terms of clustered mutations; Figure S7: Analysis of apoptotic CTCs; Table S1: Analysis of *PIK3CA* mutations by ddPCR; Table S2: *AKT1* E17K mutation on single CTCs of patient 1 during the observation period; Table S3: Analysis of *ESR1* mutations in ctDNA by ddPCR; Table S4: Primers for sequencing of the *AKT1* region including the E17 mutational hotspot; Table S5: Primers for *PIK3CA* ddPCR; Table S6: Probes for *PIK3CA* ddPCR.
