# Supplementary material for: Multiparametric Circulating Tumor Cell Analysis to Select Targeted Therapies for Breast Cancer Patients
Source: Cancers (Basel). 2021 Nov 29;13(23):6004. doi: 10.3390/cancers13236004 (PMC8657376; doi:10.3390/cancers13236004)
Supplement: Supplementary file 1 [file cancers-13-06004-s001.zip › cancers-1453191-supplementary.pdf]

Supplementary data

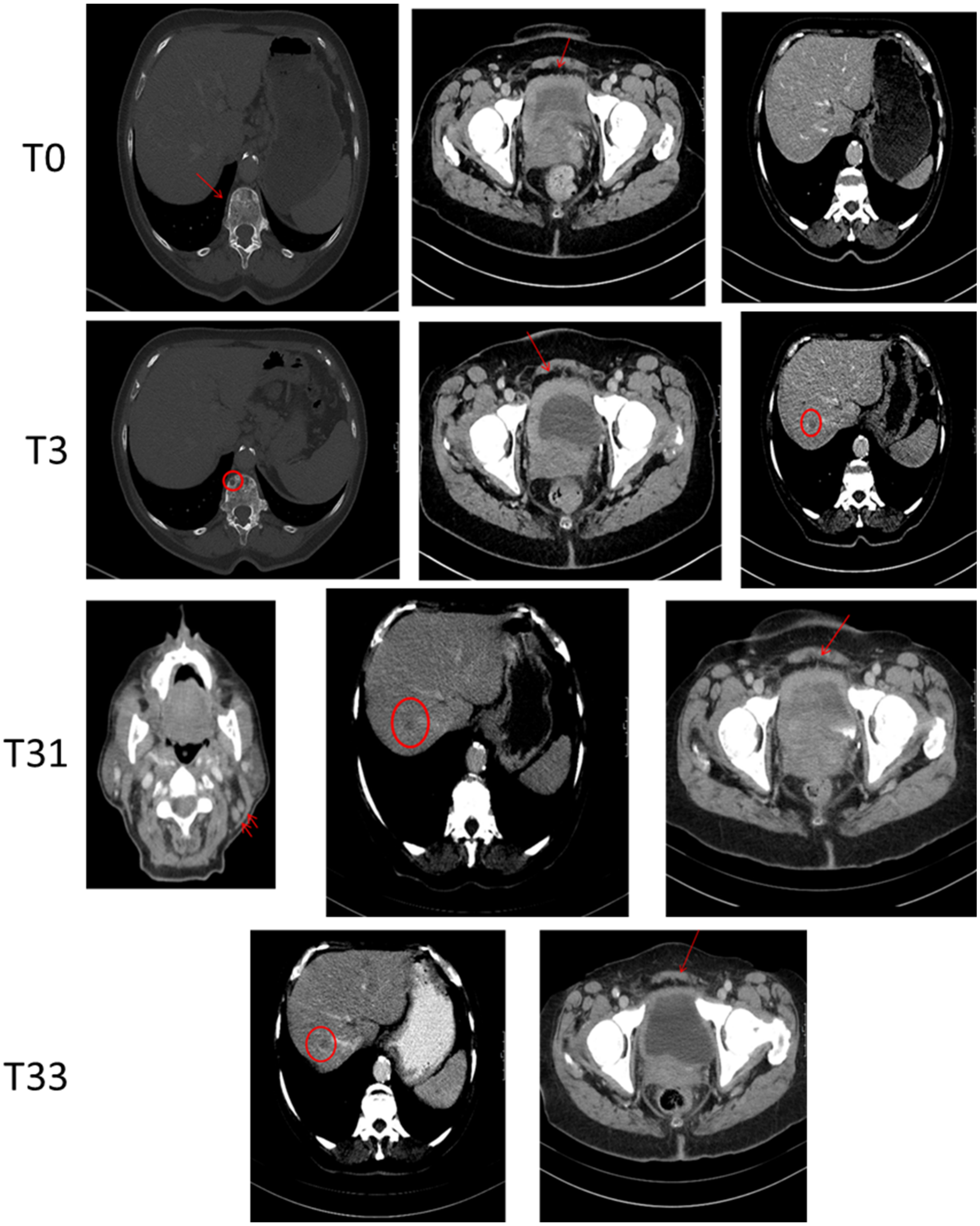

Figure S1: Staging CT analysis of patient 1

Staging CTs were performed regularly during the course of the disease. Discussed areas are highlighted with red circles and arrows.

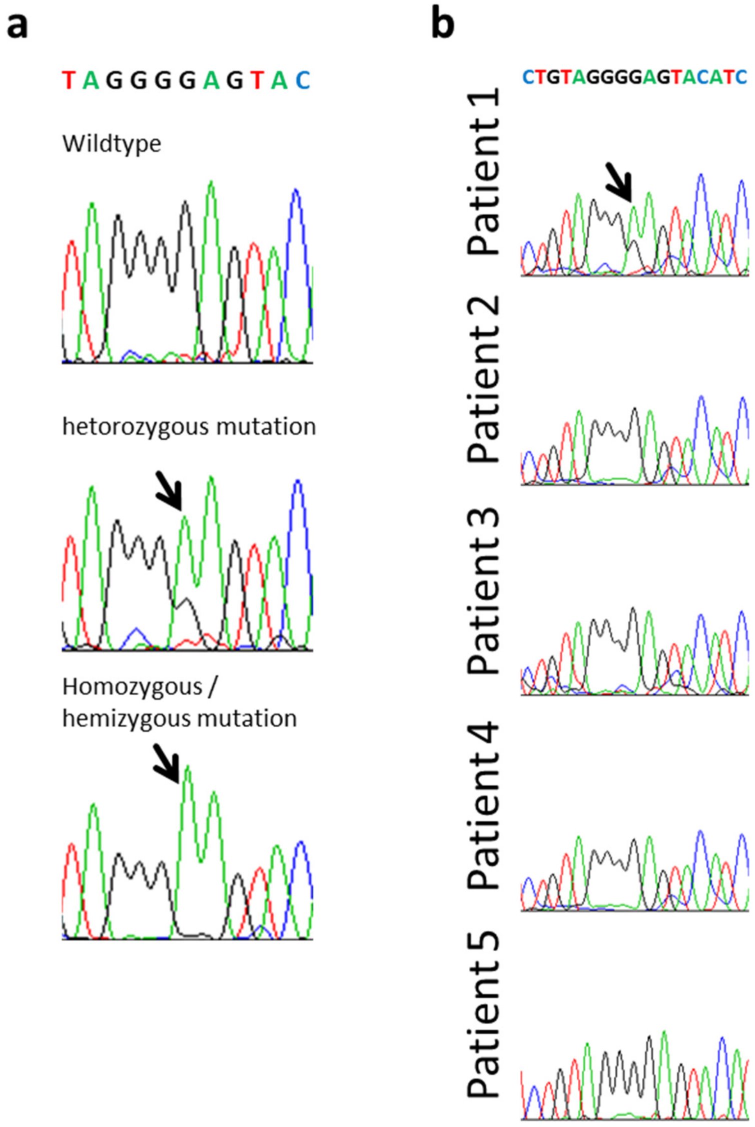

Figure S2: Mutation analysis of the *AKT1* E17K hotspot

(a) References for E17 hotspot mutation in *AKT1*. (b) Sanger sequencing results on pooled whole-genome amplification products from single CTCs. Patient 1 shows a heterozygous mutation, highlighted with an arrow.

	Gene	Variant	VAF	Treatment
Patient 1	CDH1	Q23*	47.09%	Wnt Pathway Inhibition (Pri-724)
	ERCC2	K751Q	31.53%	Radiotherapy
		D312N	43.90%	Radiotherapy
	ABCC1	G671V	45.68%	Doxorubicin
	ABCG2	Q141K	45.08%	Sunitinib
Patient 3	PIK3CA	H1047R	10.34%	PI3K Inhibition (Alpelisib + Fulvestrant)
				mTOR Inhibition (Everolimus, Sirolimus)
				HER2-inhibition (Trastuzumab)
	CHEK2	K373*	9.09%	PARP Inhibition
	SMAD4	R497H	12.00%	5-Fluorouracil
	MTHFR	A222V	52.07%	Methotrexate
	FCGR3A	F176V	44.44%	Cyclophosphamide + Doxorubicin + Prednisone + Rituximab + Vincristine
	SLCO1B1	V174A	100.00%	Methotrexate
	RARG	S427L	49.06%	Idarubicin, Daunorubicin, Anthracyclines, Epirubicin, Doxorubicin
	XPC	Q939K	100.00%	Cisplatin

Effective
Ineffective
Safety

Somatic Variant
Germline Variant

Figure S3: Whole exome sequencing of the primary tumor of patients 1 and 3

For each detected mutation, treatments and their efficacies and safety concerns are listed (effective: potentially effective treatment option; ineffective: potentially ineffective treatment; safety: potential safety concern).

Variant allele frequency (VAF)

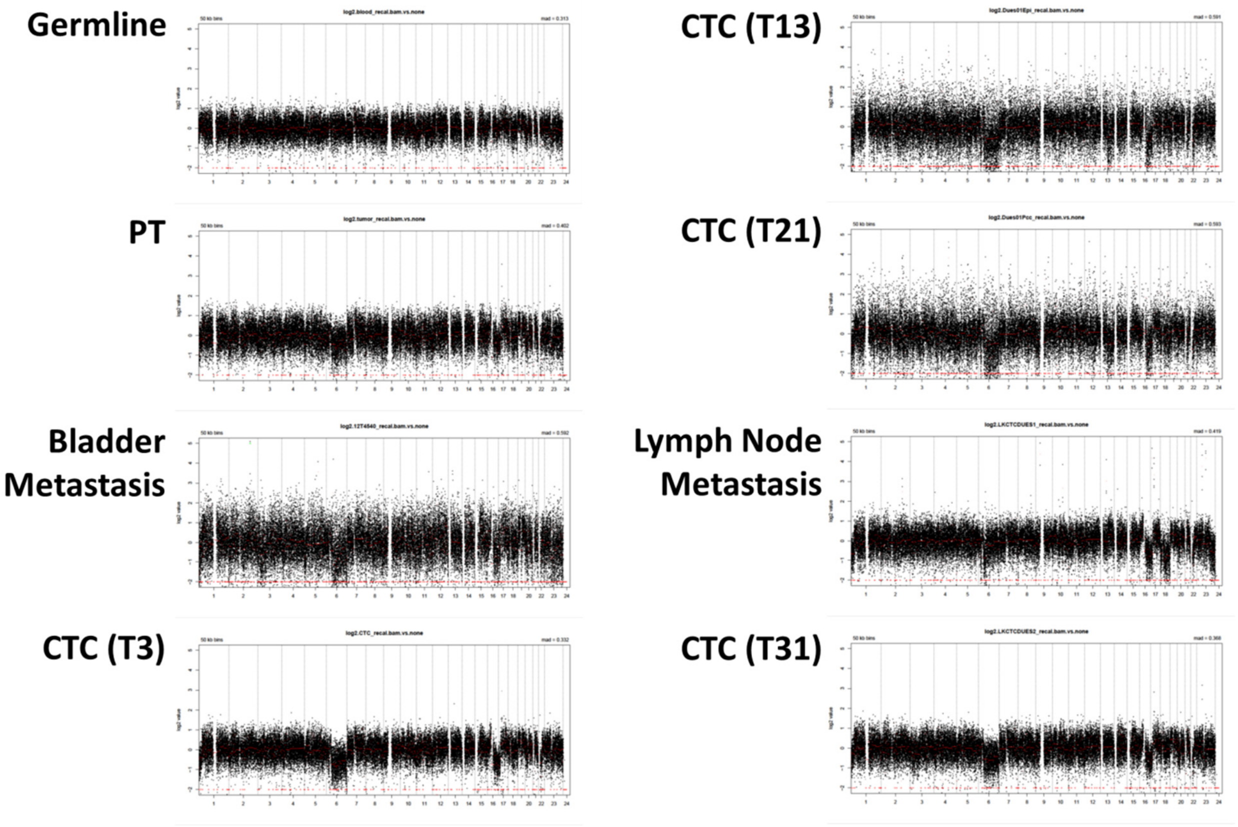

Figure S4: Copy number profiles determined by whole-exome sequencing

Primary Tumor (PT); Circulating tumor cell (CTC)

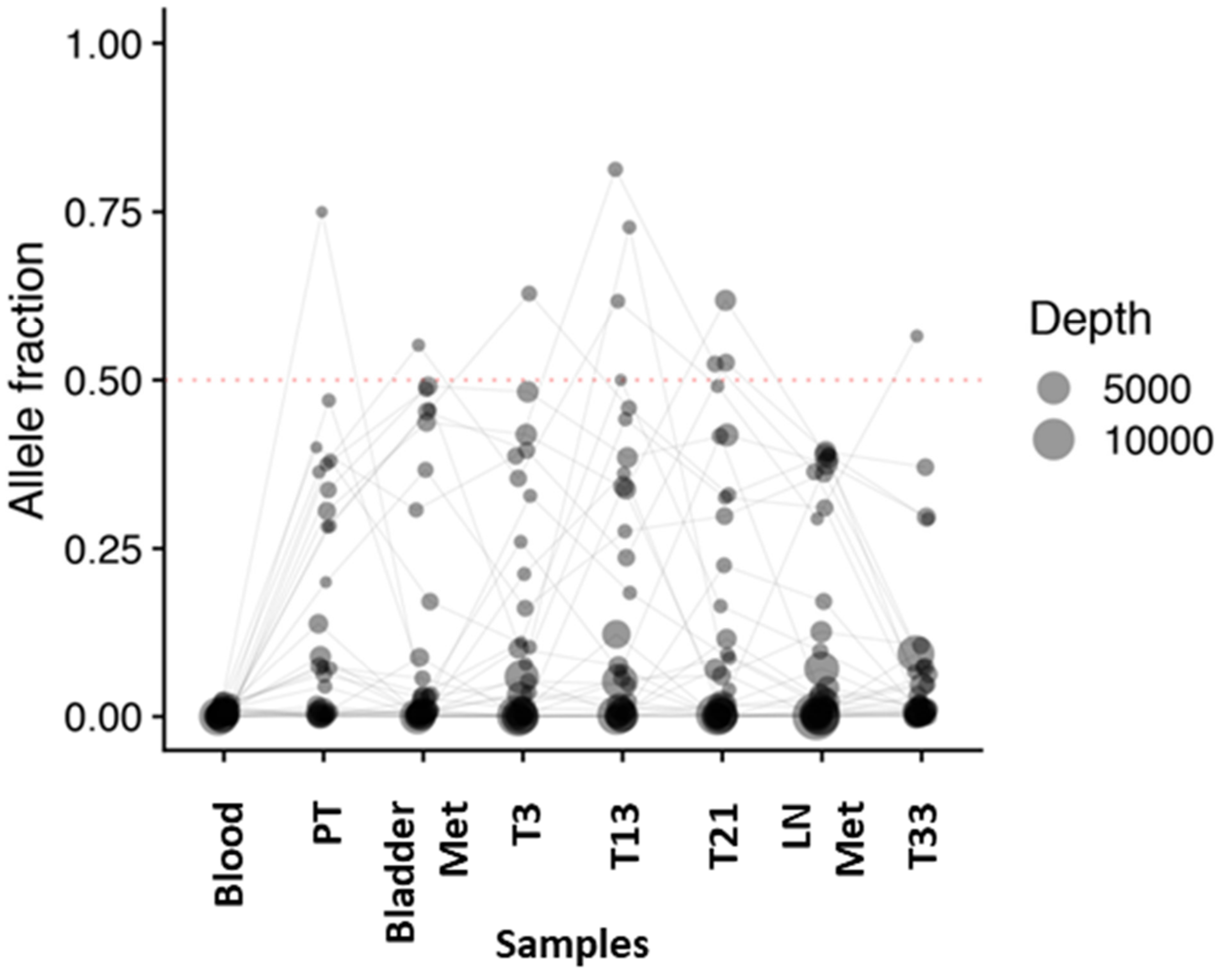

Figure S5: Allele fractions of clustered mutations

Allele fractions of clustered mutations in regions with copy number neutrality common to all samples are shown. Bulk leukocytes sorted based on their CD45 positivity served as a germline DNA control (Blood). DNA from the bladder metastasis (Met) and the lymph node (LN) Met was extracted from tissue biopsies at T0 and T31, respectively.

Primary tumor (PT)

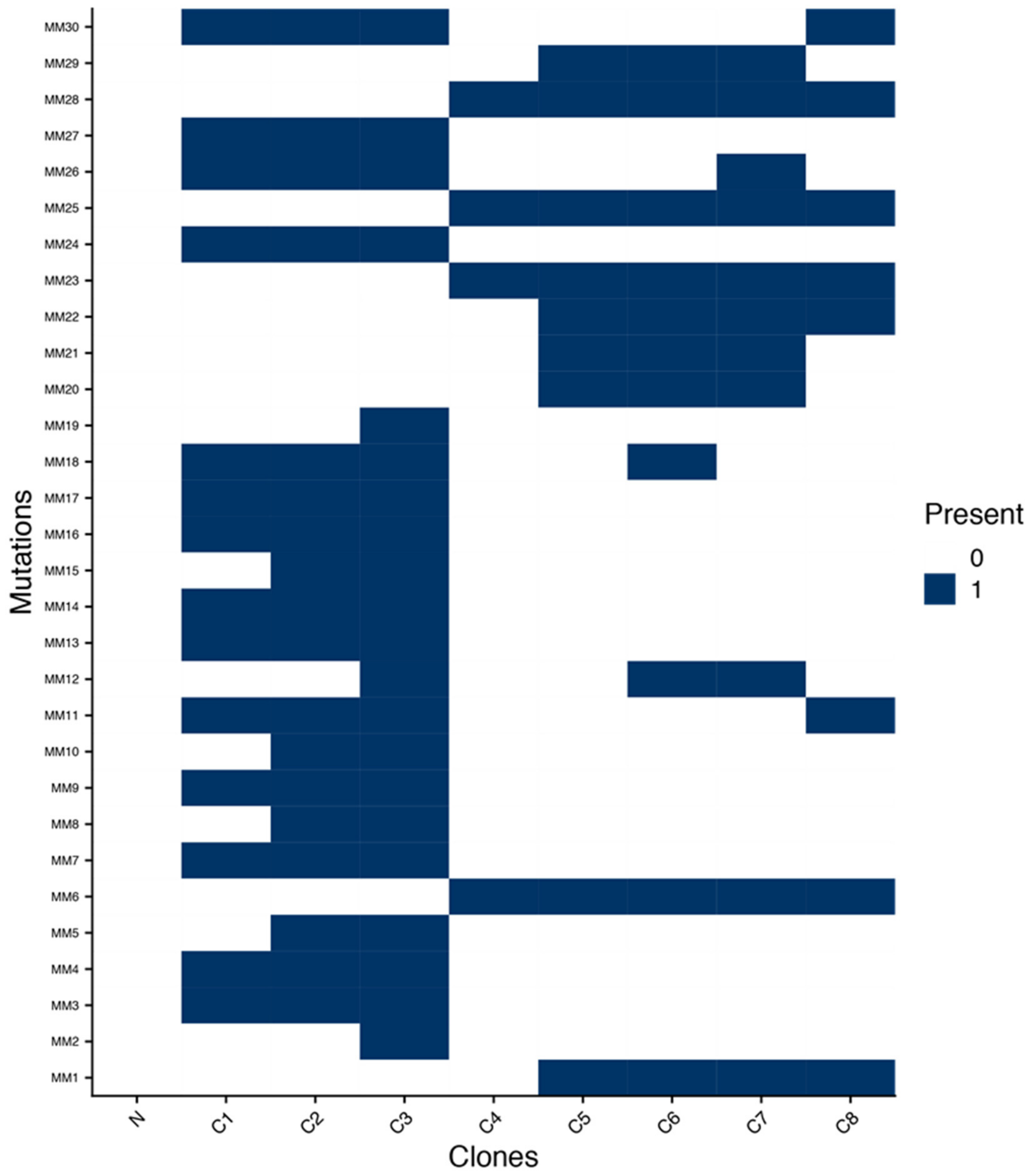

Figure S6: Genotypes of the identified tumor clones, in terms of clustered mutations

30 meta-mutations (MM) were identified and assigned to 8 tumor cell clones (C1 – C8). Mutations were absent in germline DNA (N).

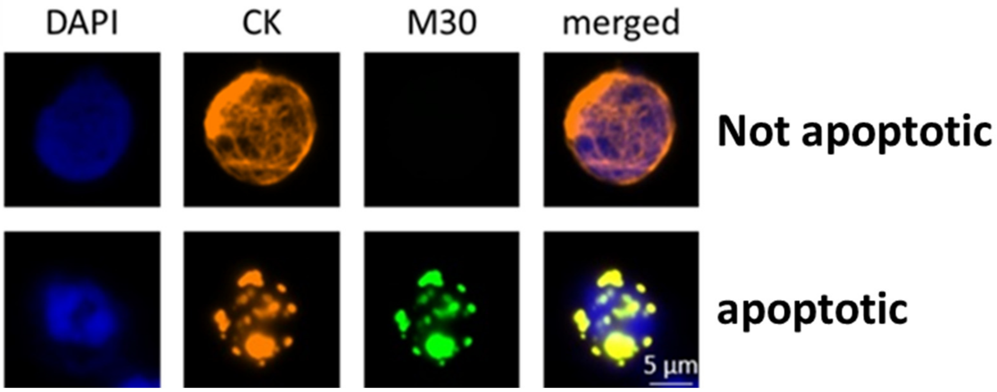

Figure S7: Analysis of apoptotic CTCs

The CTCs were stained for caspase cleaved cytokeratin (CK) 18 (M30). Positive cells were considered as apoptotic (original magnification 40×).

Table S1: Analysis of *PIK3CA* mutations by ddPCR

DNA extracted from a time matched needle biopsy of a metastasis from patients 4 and 5 was analyzed by digital droplet PCR. The expected mutations were detected in the circulating tumor cells (CTCs) of patients 3, 4 and 5. Tissue of a time matched biopsy of patient 3 was not available (na).

	**Tissue Biopsy**	**CTCs**
**Patient 3 (H1047R)**	na	100%
**Patient 4 (H1047L)**	43.1%	50.3 ± 0.4%
**Patient 5 (E545K)**	7.9% ± 6.6%	68.1% ± 4.5%

Table S2: *AKT1* E17K mutation on single CTCs of patient 1 during the observation period

AKT1 E17K mutation on single circulating tumor cells (CTCs) from different time points were analyzed by targeted Sanger sequencing. The varaint alllele frequency is shown.

Het (heterozygous)

	**T3**	**T12**	**T21**	**T31**	**T33**	**T37**
**CTC 1**	100%	0%	0%	0%	Het	100%
**CTC 2**	Het	100%	100%	0%	100%	100%
**CTC 3**	100%	0%	100%	Het	0%	0%
**CTC 4**	100%	0%	0%	0%	Het	Het
**CTC 5**	100%	100%		Het	100%	0%
**CTC 6**	100%	100%		100%	0%	100%
**CTC 7**	0%	Het		0%	Het	0%
**CTC 8**	Het					100%
**CTC 9**	100%					100%

Table S3: Analysis of *ESR1* mutations in ctDNA by ddPCR

“-“ indicates that no mutation was detected

	**E380**	**Y537**	**D538**
**T3**	0.2%	-	-
**T13**	-	-	-
**T21**	-	-	-

Table S4: Primers for sequencing of the *AKT1* region including the E17 mutational hotspot

	**Sequence**
**Forward external**	5' CACATCTGTCCTGGCACACC 3'
**Forward internal**	5' AGTTCCTGCCTGGCTGCCTG 3'
**Reverse**	5' GCCGCTCCTTGTAGCCAATG 3'

Table S5: Primers for *PIK3CA* ddPCR

	**Sequence**
**H1047R forward**	5′ GCAAGAGGCTTTGGAGTATTTCATG 3′
**H1047R reverse**	5′ GCTGTTTAATTGTGTGGAAGATCCAA 3′
**H1047L forward**	5′ GCAAGAGGCTTTGGAGTATTTCATG 3′
**H1047L reverse**	5′ GCTGTTTAATTGTGTGGAAGATCCAA 3′
**E545K forward**	5′ TCAAAGCAATTTCTACACGAGATCCT 3′
**E545K reverse**	5′ CTGTGACTCCATAGAAAATCTTTCTC 3′
**E542K forward**	5′ GGGAAAATGACAAAGAACAGCTCAA 3′
**E542K reverse**	5` CTGTGACTCCATAGAAAATCTTTCTCCT 3′

Table S6: Probes for *PIK3CA* ddPCR

The following TaqMan probes with a 5′ fluorophore and a 3′ non-fluorescent quencher (NFQ) were utilized

	**Sequence**
**H1047R Wild type**	5′ (HEX)-CCACCATGATGTGCATC-(MGB NFQ) 3′
**H1047R Mutant**	5′ (FAM)-CACCATGACGTGCATC-(MGB NFQ) 3′
**H1047L Wild type**	5′ (HEX)-CCACCATGATGTGCATC-(MGBNFQ) 3′
**H1047L Mutant**	5′ (FAM)-CACCATGAAGTGCATC-(MGB NFQ) 3′
**E545K Wild type**	5′ (HEX)-CTCTCTGAAATCACTGAGCAG-(MGB NFQ) 3′
**E545K Mutant**	5′ (FAM)-CTCTGAAATCACTAAGCAG-(MGB NFQ) 3′
**E542K Wild type**	5′ (HEX)-CCTCTCTCTGAAATCA-(MGB NFQ) 3′
**E542K Mutant**	5′ (FAM)-CCTCTCTCTAAAATCA-(MGB NFQ) 3′
